# Kaempferol Reduces Cardiopulmonary Load and Muscular Damage in Repeated 400‐m Sprints: A Double‐Blind, Randomized, Placebo‐Controlled Trial

**DOI:** 10.1002/fsn3.4506

**Published:** 2024-10-14

**Authors:** Koichi Okita, Tsubasa Mizokami, Minoru Akiyama, Yasutaka Ikeda

**Affiliations:** ^1^ Hokusho University Graduate School of Lifelong Sport Ebetsu Hokkaido Japan; ^2^ Saga Nutraceuticals Research Institute Otsuka Pharmaceutical Co., Ltd. Kanzaki‐gun Saga Japan; ^3^ Otsu Nutraceuticals Research Institute Otsuka Pharmaceutical Co., Ltd. Otsu Shiga Japan

**Keywords:** athlete, ergogenic aid, exercise performance, flavonoid, highland, kaempferol

## Abstract

Plants exposed to hypoxic conditions have been suggested to produce more biologically active phytochemicals than those exposed to normal oxygen levels. Previously, we investigated 314 highland crop species and showed that the flavonoid kaempferol extracted from a highland quinoa grain markedly increased mitochondrial metabolism and ATP production in a hypoxic environment in vitro. Thus, we hypothesized that kaempferol would be effective during exercise under harsh conditions, in which anaerobic metabolism occurs. This study adopted a double‐blind, placebo‐controlled, crossover design to investigate the effect of a single oral dose of kaempferol (10 mg) on the athletic performance‐related indicators of 13 male university athletes (20.8 ± 0.7 years) who performed two consecutive 400‐m runs with the shortest 90‐min interval assuming qualifying and main races. Although no significant differences were observed in the 400‐m race times between the placebo and kaempferol groups, kaempferol intake markedly reduced the respiratory and heart rates during the second run (*p* < 0.05). In addition, kaempferol intake reduced the levels of muscle damage markers, myoglobin, and aspartate transaminase (*p* < 0.05). A single oral dose of kaempferol reduced the cardiopulmonary burden and muscle damage in individuals participating in 400‐m runs. Kaempferol may be a useful supplement for relieving the physical load, particularly in individuals performing strenuous exercises with high oxygen demand.

**Trial Registration:** UMIN Clinical Trials Registry in Japan: UMIN000049588

## Graphical abstract

A single oral dose of kaempferol reduced cardiopulmonary burden and muscle damage in individuals participating in 400‐m runs. Kaempferol may serve as a useful supplement for relieving the physical load, particularly in individuals performing strenuous exercise with a high oxygen demand.

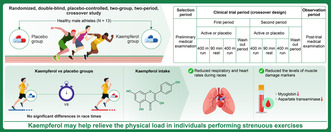

## 1 Introduction

Plants biosynthesize phytochemicals in response to environmental stresses (Dinkova‐Kostova 2008; Zaynab et al. 2018). For instance, flavonoids and carotenoids are synthesized in plants to quench or scavenge the free radicals formed during UV light exposure (Dinkova‐Kostova 2008). Flavonoids possess polyphenolic structures, exhibit various health‐promoting properties (Luo et al. 2019), exert free radical‐scavenging effects, have anti‐fatigue effects, and enhance physical strength through strong antioxidant activities (Dinkova‐Kostova 2008; Luo et al. 2019) and the nitric oxide (NO) signaling pathway (d'Unienville et al. 2021; Rizza et al. 2011).

Previously, we showed that plants exposed to hypoxic conditions produce more biologically active phytochemicals than those exposed to normal oxygen levels (Mizokami, Akiyama, and Ikeda 2021). Specific components of these plants may help improve highland residents' health and physical strength. We focused on the components of plants primarily cultivated in highland areas (Mizokami, Akiyama, and Ikeda 2021). Screening tests for 65 reference phytochemicals from 314 crop species conducted in a hypoxic cellular evaluation system resembling a biological environment indicated that kaempferol had the strongest activity, which markedly increased the intracellular ATP content by activating mitochondrial metabolism (Akiyama et al. 2022). Kaempferol is a natural flavonoid in various vegetables, fruits, and beverages as a glycoside (Calderon‐Montano et al. 2011; Mizokami, Akiyama, and Ikeda 2021). Moreover, it exhibits strong antioxidant capacity (Singh et al. 2008; Tzeng et al. 2011), and has been experimentally proven to possess anti‐inflammatory, anticancer, cardioprotective, and neuroprotective properties (Chen and Chen 2013; Imran et al. 2019; Wang et al. 2018; Yao et al. 2019).

In this study, we hypothesized that kaempferol, abundant in high‐altitude food, may support successful high‐altitude dwellers' physical capabilities and daily health. During strenuous exercise, the oxygen demand in skeletal muscles increases, and insufficient oxygen supply and hypoxia suppress mitochondrial function and impair athletic performance. Exhaustive exercise increases the production of reactive oxygen species (ROS), leading to muscle fiber damage, which eventually results in muscle fatigue (Peternelj and Coombes 2011). If kaempferol enhances mitochondrial efficiency in vivo and has vigorous antioxidant activity, it could improve athletic performance by reducing cardiopulmonary responsiveness and muscle damage during vigorous exercise. However, no studies have investigated the effects of kaempferol on exercise or athletic performance. Therefore, we investigated the impact of a single oral dose of kaempferol (10 mg) on performance‐related indicators in repeated 400‐m runs in a randomized, double‐blind, placebo‐controlled crossover study.

## 2 Materials and Methods

### 2.1 Ethical Declarations

The study protocol complied with the principles of the Declaration of Helsinki. This study was approved by the Research Ethics Committee of Hokusho University (2017‐009) and the Ethics Review Board of Otsuka Pharmaceutical Research Institute (1707). The study was registered in Japan's UMIN Clinical Trials Registry (UMIN000049588). All participants received a full explanation of the purpose, methods, safety, and other aspects of the study before participating, and all provided written informed consent.

### 2.2 Participants, Eligibility Criteria, and Randomization

**FIGURE 1:**
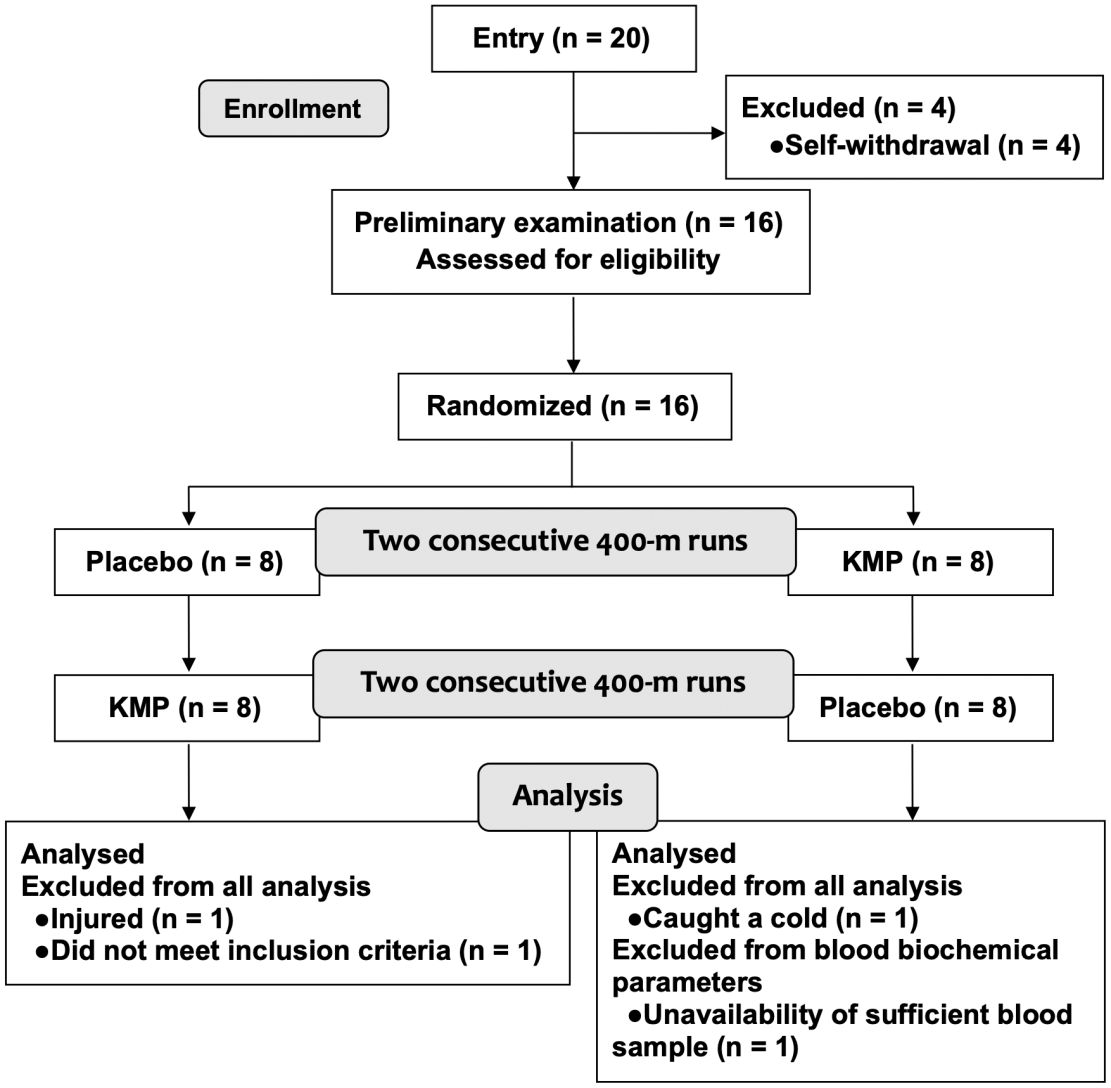
CONSORT flow diagram of a crossover design of a randomized, double‐blind, placebo‐controlled study.

This study was conducted with local students at an athletics stadium in Ebetsu City, 40 m above sea level. We recruited healthy male college athletes from a physical education course who were familiar with a 400‐m run. During the preliminary examination, a subject background and medical questionnaire were completed by the participants; basic measurements (height, weight, blood pressure, and pulse rate) were recorded, and blood (for blood biochemical and hematological tests) and urine sampling (for general urine test) were performed. The following participants were excluded: those whose laboratory test values deviated from the reference range; those with disorders of the digestive, circulatory, or endocrine systems; those who regularly used drugs and supplements to treat diseases; and those who may have undergone doping tests. A physician assessed the overall health status of participants. Sixteen participants were included in the study. Subsequently, a person blinded to this study's conduct and analysis randomly assigned the participants to two groups and two periods (eight cases per group) using the Latin square method. However, three participants were excluded during the study period because they were injured or contracted a cold, and one participant was excluded because of insufficient blood samples. Eventually, 13 of the 16 participants were included in the athletic performance analysis and 12 of the 16 participants were included in the blood biochemical parameter analysis (Figure 1).

### 2.3 Test Supplement Ingestion and Blinding

The investigator ensured that the two capsule types were indistinguishable. The relevant identification codes were printed on a pack containing each substance, sealed in an envelope, and delivered to the outsourcing provider. The identification codes were sealed in an envelope and sent to the allocator, which confirmed that the capsule could not be identified based on appearance or smell. The allocator replaced the kaempferol (active) supplement and placebo codes with control codes that could not be easily identified, thereby blinding the study. The products were then transferred to the test product manager. The control codes were tightly sealed in an envelope together with the corresponding table and remained with the allocator until the study was unblinded.

**FIGURE 2:**
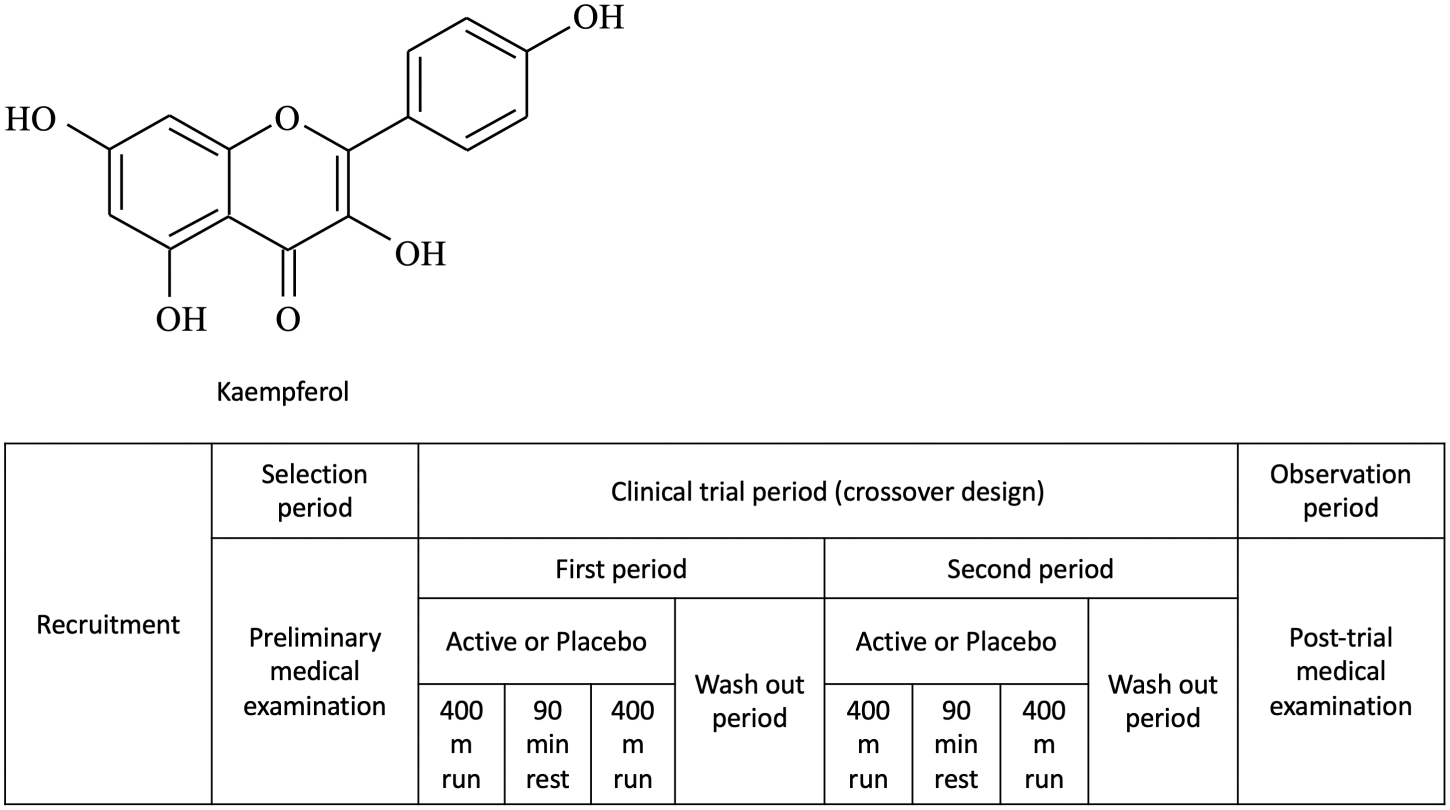
Structure of the flavonoid kaempferol and schematic of a crossover design of a randomized, double‐blind, placebo‐controlled study.

The structure of kaempferol is shown in Figure 2. The test supplement was administered as a capsule with the placebo, substituting kaempferol with cornstarch. Kaempferol glycosides from quinoa powder were converted to aglycones via aromatase treatment. The active and placebo capsules had the same mass and contained similar amounts of nutrients and calories. On each testing day, the active capsule (containing 10 mg kaempferol) or placebo capsule (containing < 0.001 mg kaempferol) was ingested in a randomly assigned order, and both capsules were ingested after fasting in the morning. Each capsule was swallowed in a single dose, and the subsequent test capsule was ingested 3 days or more after the previous intake to ensure sufficient wash‐out, confirmed by preliminary laboratory examination. Otsuka Pharmaceutical Co. Ltd. (Tokyo, Japan) provided the capsules used in this study. The participants and researchers were blinded after being assigned to the interventions by an independent test food allocation manager. In preliminary studies, the effect of kaempferol intake on exercise capacity plateaued between 10 and 50 mg. Based on these results, 10 mg was administered in the current study. The safety of kaempferol in humans has been demonstrated in clinical trials (Akiyama et al. 2023).

### 2.4 Experimental Design

We designed repeated 400‐m runs, assuming qualifying and main races at 90‐min intervals (officially the shortest). A randomized, double‐blind, placebo‐controlled, two‐group, two‐period crossover study is shown in Figure 2.

An ultra‐compact multichannel biometric monitor (Equivital EQ02 LifeMonitor; Hidalgo, Cambridge, UK) was attached to each participant 3 h after ingestion of the test supplement, following which the participants sprinted 400 m in real time, and their heart and respiratory rates were measured and re‐sprinted after a 90 min interval. For the 400‐m runs, a video was recorded from the center of the ground, and the time, stride width, and pitch were calculated based on equally spaced landmarks. Before and after each 400‐m run, the degree of fatigue was measured based on the visual analog scale (VAS) and the rate of perceived exertion (RPE) using the Borg scale (Borg 1982).

Blood was collected before and after the first and second 400‐m runs and 3 h after completing the second 400‐m run. The collected blood was used for plasma kaempferol concentration measurements, hematological examinations, and blood biochemical examinations. Urine was stored for up to 24 h after ingesting the test capsule, and the absorbed kaempferol content was measured. After the repeated 400‐m run test, a rest period of ≥ 3 days was provided, the test capsule was changed, and the test was repeated.

### 2.5 Biochemical Analysis

Blood was collected from the anterior elbow vein using a disposable needle, adapter, and vacuum blood collection tube. Overall, 90 mL of blood was collected, with 10 mL collected in the pretest period, 10 mL collected in the post‐test period, and 70 mL collected at each time point (aforementioned). General biochemical and hematological parameters were measured during pre‐ and post‐medical examinations to evaluate the health condition of each participant.

During the run test, myoglobin, creatine phosphokinase (CPK), aspartate transaminase (AST), alanine aminotransferase (ALT), and lactate dehydrogenase (LDH) concentrations were measured using a chemiluminescence immunoassay and the UV method (SRL, Inc. Tokyo, Japan). Lactate and pyruvate levels were measured using an enzymatic method after rapid centrifugation at 3000 rpm for 5 min. Kaempferol concentrations in the plasma and urine were measured at Otsuka Pharmaceutical Co., Ltd. The samples were deconjugated using β‐glucuronidase and extracted using solid‐phase extraction. The extracted samples were analyzed quantitatively using liquid chromatography‐mass spectrometry (Nexera X2; Shimadzu, Kyoto, Japan). The total urinary kaempferol concentration and volume were measured and calculated using the following formula:

$$\begin{array}{c} \text{Urinary kaempferol excretion}\;\left(\mathrm{mg}\right)\\ =\text{total urinary kaempferol concentration}\;\left(\mathrm{\mu M}\right)\\ \times \text{urinary volume}\;\left(L\right)\times 286.24\;\left(\text{molecular weight of kaempferol}\right)\\ \times 1/1000 \end{array}$$

The collected urine and container were weighed using a weighing scale, and the difference between the weights of the container with urine and the empty containers was considered as the urine weight. The urine weight was multiplied by the specific gravity to obtain the urine volume.

### 2.6 Statistical Analysis

Variables are expressed as the mean ± standard deviation (SD) or standard error (SE). Statistical analyses were performed using SAS software (version 9.4; SAS Institute, Cary, NC, USA), and statistical significance was determined at *p* < 0.05. Changes arising from different treatments were assessed using a linear mixed‐effects model with fixed terms fitted for treatment, period, and sequence and a random effect for subject‐within‐sequence. As we used a mixed model of the repeated‐measures approach, missing data were not included in the primary model. In addition to the per‐protocol analysis, we conducted a sub‐analysis using the median value of urinary kaempferol excretion levels (stratified analysis). Based on previous studies investigating the effects of nutritional components on exercise performance, the minimum necessary sample size to observe the impacts was nine (Bailey et al. 2010; Kelly et al. 2013; Larsen et al. 2007). Regarding athletic performance, a previous study using a similar crossover design to examine the effects of test meals on 400‐m sprints observed significant differences in race times in 10 (Caciano et al. 2015) or 11 participants (Limmer, Eibl, and Platen 2018). Besides, an a priori calculation indicated that 11 participants were needed to detect significant differences in blood samples in 400‐m sprints, based on an estimated *α* level of 0.05 and a power of 95% (Caciano et al. 2015; Limmer, Eibl, and Platen 2018). We were able to analyze 13 participants, which was more than in those studies.

## 3 Results

Table 1 lists basic participant data. Sufficient increases in blood kaempferol concentration were observed during the races (Table S1). Table S2 shows the participants' 24‐h urinary kaempferol excretion and excretion rates.

**TABLE 1:** Participant characteristics.

Parameters	Numerical values
*N*	13
Age (years)	20.8 ± 0.7
Height (cm)	174.9 ± 6.1
Weight (kg)	66.5 ± 5.0
Body mass index (kg·m^−2^)	21.7 ± 1.4
Maximum plasma kaempferol concentration (μM)	0.274 ± 0.066
Urinary KMP excretion (mg)	0.336 ± 0.194

*Note:* Mean ± SD.

Contrary to expectations, there were no significant differences between the 400‐m race times when different supplements were administered, whereas the second 400‐m race time in the kaempferol group was significantly lower than the first 400‐m race time (Table 2). The stride width and pitch values for every 50 m during the 400‐m races did not show significant differences (Tables S3 and S4, respectively).

**TABLE 2:** The 400‐m race times.

Group	400‐m race time (s)	
	1st run	2nd run
Placebo	59.62 ± 1.40	59.51 ± 1.45
Active	60.47 ± 1.37	59.43 ± 1.39*

*Note:* Active refers to 10 mg kaempferol‐containing capsules. Mean ± SE. Mixed model for crossover design.

*p < 0.05 vs. 1st run (*p* = 0.0146).

**FIGURE 3:**
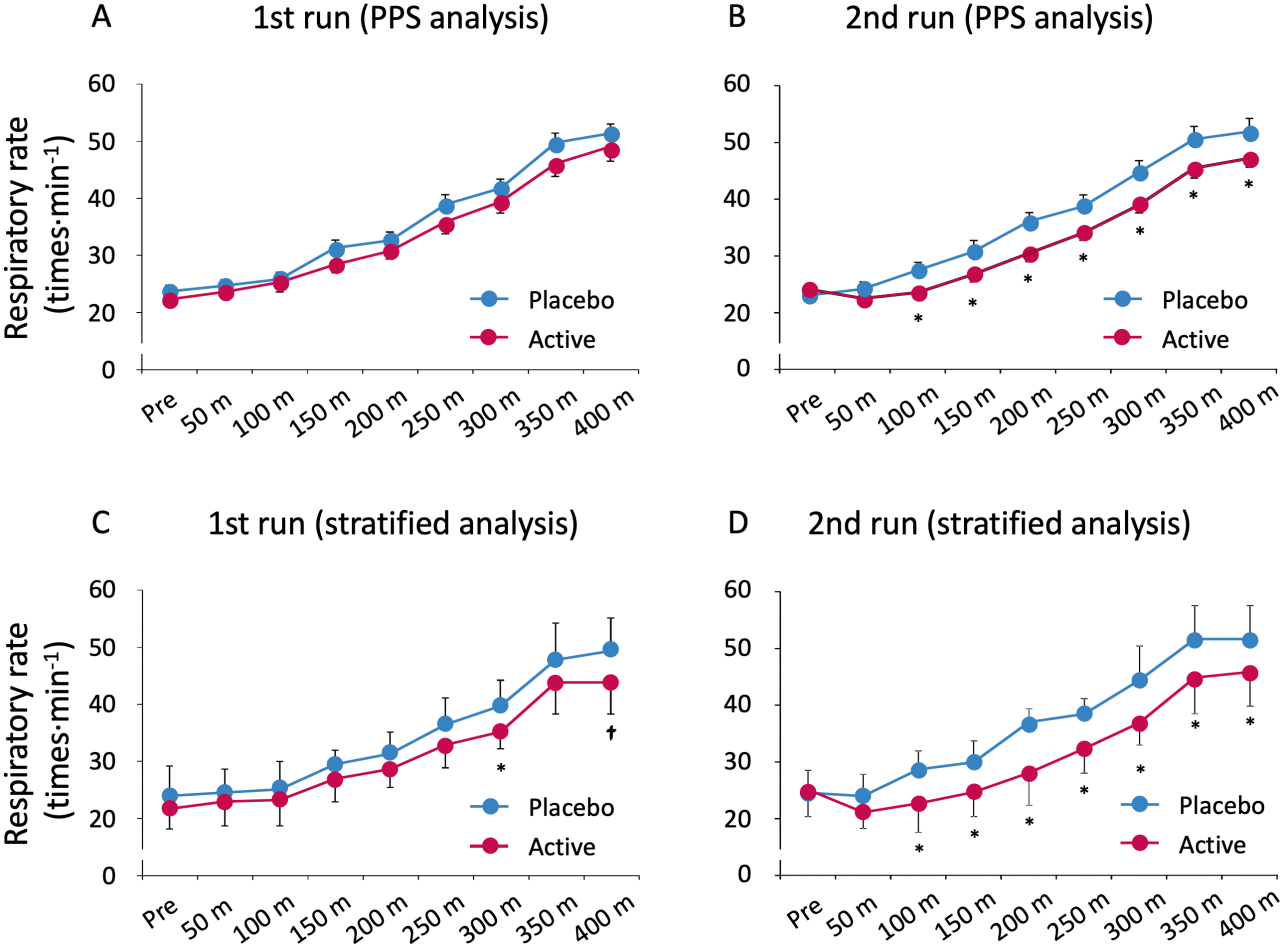
Respiratory rate at each point during the 400‐m races. Active means a 10 mg kaempferol‐containing capsule. No significant difference was observed between the respiratory rates in the two groups in the first run (A). However, there was a significant reduction in the respiratory rate from the 100‐m point to the endpoint in the second run in the kaempferol intake group compared to that in the placebo group (B). In the stratified analysis, the respiratory rate in the kaempferol intake group decreased in the first run (C) and significantly decreased in the second run compared to that in the placebo group (D). PPS, per protocol set. Stratification criteria are defined as above the median level of urinary kaempferol excretion (*n* = 7). Data are expressed as mean ± SE (^†^ *p* < 0.1, *p < 0.05, vs. placebo).

Table 3 shows the total number of respirations during each 400‐m race. Kaempferol intake significantly lowered total respiration in the second run compared with the placebo. Figure 3 shows the respiratory rate at each point during the 400‐m race. In the first run, the two groups had no significant difference in respiratory rates (Figure 3A). However, in the second run, kaempferol intake significantly decreased the respiratory rate compared to the placebo from the 100‐m point to the endpoint at 400 m (Figure 3B). Furthermore, in the stratified analysis, the respiratory rate in the kaempferol‐treated group tended to decrease in the first run (Figure 3C) and was more significant in the second run than in the placebo group (Figure 3D).

**TABLE 3:** Total number of respirations during each race.

Group	Total number of respirations (times/race)	
	1st run	2nd run
Placebo	37.8 ± 1.6	39.0 ± 1.8
Active	35.6 ± 1.4	34.4 ± 1.2*

*Note:* Active refers to 10 mg kaempferol‐containing capsules. Mean ± SE. Mixed model for crossover design.

*p < 0.05 vs. placebo (*p* = 0.0140).

**FIGURE 4:**
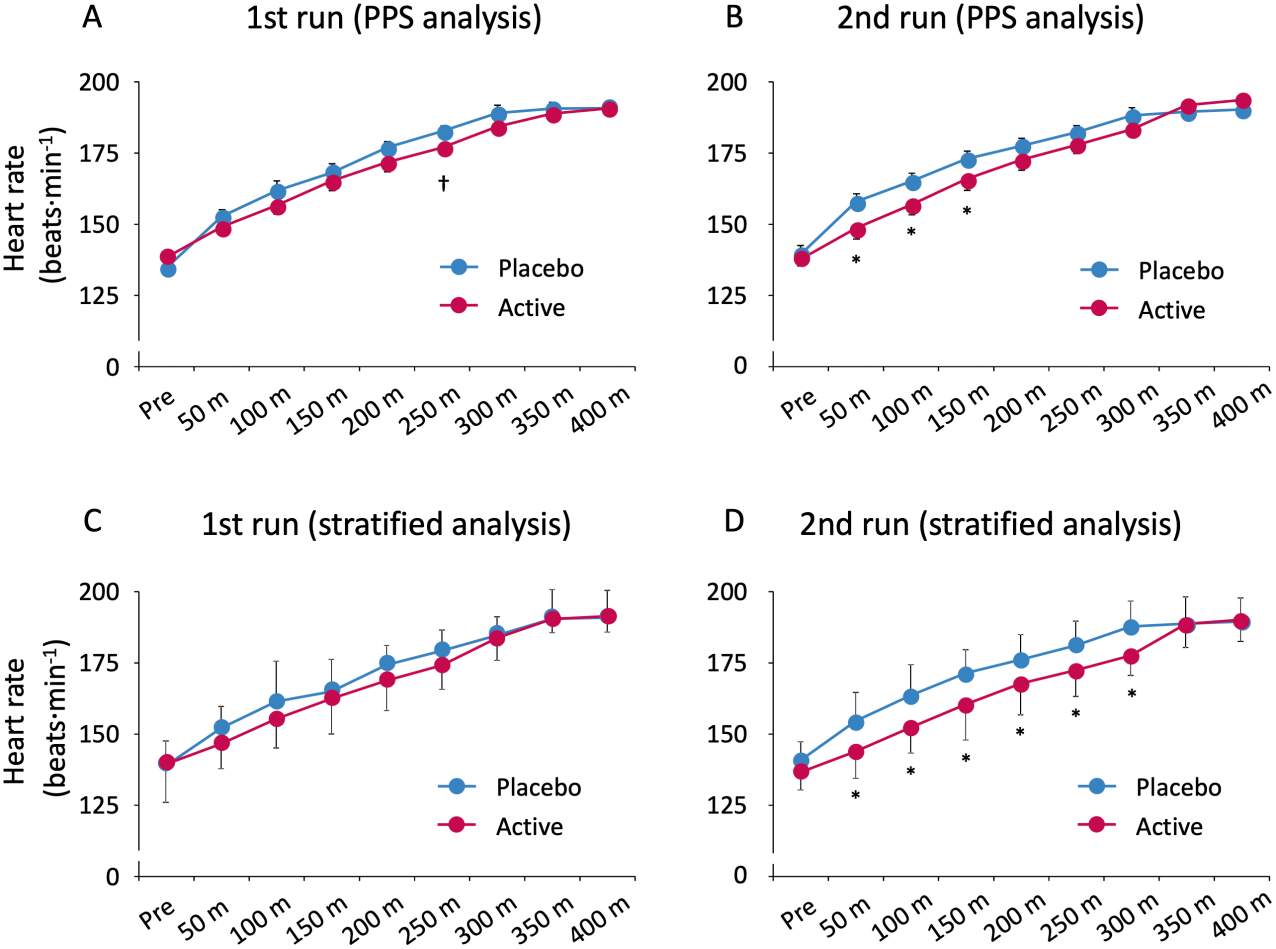
Heart rate at each point during the 400‐m races. No significant difference was observed between the heart rates in the two groups in the first run (A,C); however, in the second run, the heart rate in the kaempferol intake group decreased significantly compared to that in the placebo group at the 50–150‐m point in the PPS analysis (B). In the stratified analysis, the heart rate in the kaempferol intake group decreased more significantly than that in the placebo group at the 50–300‐m point in the second run (D). PPS, per protocol set. Stratification criteria are defined as above the median level of urinary kaempferol excretion (*n* = 7). Data are expressed as mean ± SE (^†^ *p* < 0.1, *p < 0.05, vs. placebo).

Heart rate was not significantly different between the two groups in the first run (Figure 4A,C); however, in the second run, a significant decrease was observed in heart rate at the 50–150‐m point with kaempferol intake compared to placebo intake (Figure 4B). In the stratified analysis, the heart rate decreased significantly at the 50–300‐m point in the second run with kaempferol intake than with placebo intake (Figure 4D).

No significant differences were observed in the VAS and RPE values between the placebo and kaempferol groups (Table 4).

**TABLE 4:** Perceived exhaustion.

Variables	Group	Numerical values	
		1st run	2nd run
VAS (cm)	Placebo	7.8 ± 0.3	7.2 ± 0.5
	Active	8.2 ± 0.4	7.9 ± 0.3
RPE	Placebo	16.9 ± 0.6	16.6 ± 0.5
	Active	17.5 ± 0.4	16.9 ± 0.5

*Note:* RPE, rate of perceived exertion according to the Borg scale. Active refers to 10 mg kaempferol‐containing capsules. Mean ± SE.

Abbreviation: VAS, visual analog scale.

**FIGURE 5:**
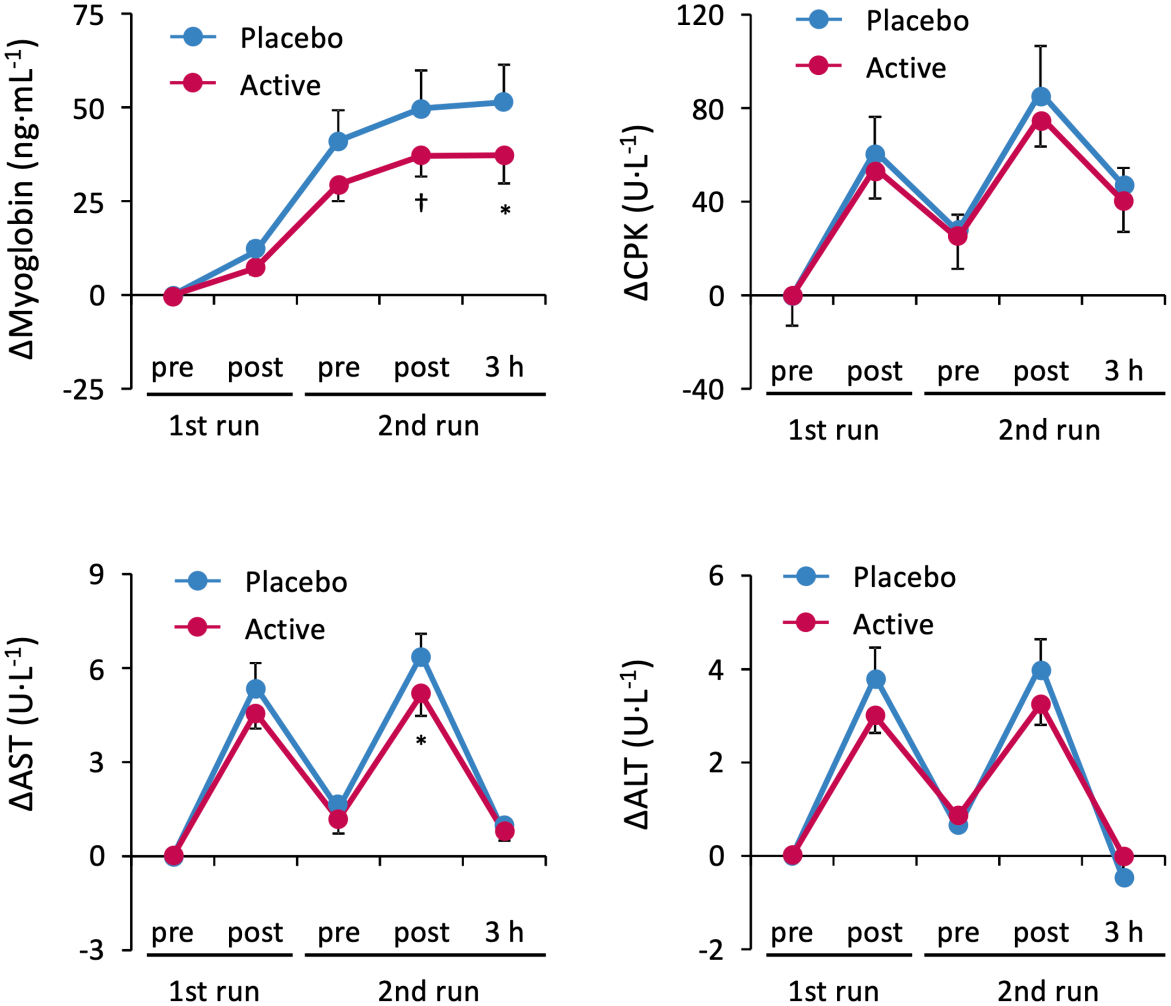
Changes in the blood marker levels for muscle damage (myoglobin, CPK, AST, and ALT) during the 400‐m races. Active means a 10 mg kaempferol‐containing capsule. Significant reductions were noted 3 h after the second run for myoglobin and immediately after the second run for AST in the kaempferol intake group compared to that in the placebo group. ALT, alanine aminotransferase; AST, aspartate transaminase; CPK, creatine phosphokinase. Data are expressed as mean ± SE (^†^ *p* < 0.1, *p < 0.05, vs. placebo).

The absolute values of exercise metabolism and muscle damage markers are represented in Tables S5 and S6. No differences were observed in the increase in blood markers of exercise metabolism (lactate and pyruvate) before and after each race between the placebo and kaempferol intake groups (Figure S1). However, the increase in myoglobin levels, a muscle damage marker, was significantly suppressed after the second run of kaempferol treatment compared to the placebo intake (Figure 5). In addition, the increase in AST levels was blunted considerably after the second run of kaempferol treatment compared to placebo intake. No significant differences were observed in the CPK, ALT, or LDH levels between the intake groups at any of the sampling points (Figure 5, Table S6).

## 4 Discussion

Although acute oral intake of kaempferol did not shorten race time, we demonstrated that kaempferol intake improved athletic performance‐related indicators, cardiopulmonary burden, and muscular damage in individuals performing ultra‐high‐intensity field exercises. Furthermore, this effect was not accompanied by deterioration in anaerobic metabolism to lactate and pyruvate levels. The reason for this is unclear; however, the apparent reduction in respiratory and heart rates during exercise may be attributed to improved oxygen transport, utilization, and/or mechanical efficiency induced by kaempferol. These results suggest that kaempferol may be a beneficial supplement for athletes, although it did not shorten race time in the current study.

First, the reduction in cardiopulmonary burden during exercise is presumed to be due to an improvement in mitochondrial efficiency (oxygen availability), as shown in vitro (Akiyama et al. 2022; Mizokami, Akiyama, and Ikeda 2021). Although the detailed mechanism of action of kaempferol could not be elucidated from the findings of this study, its effects may be partly attributed to its strong ability to eliminate ROS (Singh et al. 2008; Tzeng et al. 2011), direct free radical scavenging, the activation of antioxidant enzymes, and the inhibition of ROS‐generating enzymes, which are common effects of several flavonoids (Dinkova‐Kostova 2008; Luo et al. 2019; Zaynab et al. 2018). In an experimental study, the antioxidant tempol improved mitochondrial oxygen utilization efficiency via NO availability, suppressing wasteful O_2_ consumption and enhancing exercise capacity. This was accompanied by reduced peak oxygen uptake in mouse models in which superoxide dismutase was knocked out, and excess ROS was produced (Kinugawa et al. 2005). If the same exercise can be performed with less oxygen consumption, it is thought that the cardiopulmonary responsibility will be reduced. Further studies are needed to clarify these issues.

Polyphenols such as flavonoids act as potent antioxidants and activate the NO signaling pathway (d'Unienville et al. 2021; Serreli and Deiana 2023). Experiments have shown that polyphenols may enhance NO production by increasing endothelial NO synthase (eNOS) expression and activity and promoting NO bioavailability via their antioxidant effects, thereby protecting NO from breakdown by ROS (Stoclet et al. 2004). NO is ubiquitous in numerous physiological processes that improve exercise performance (Jones et al. 2021; Pawlak‐Chaouch et al. 2016). Dietary nitrate intake, a donor of NO, reduces oxygen demand/cost during exercise (Bailey et al. 2010; Jones 2014; Larsen et al. 2011). Improvements in mitochondrial efficiency due to dietary nitrate intake, which has been suggested to reduce proton leaks/slippage and improve thermodynamic‐energetic coupling, have been demonstrated (Bailey et al. 2010; Jones 2014; Larsen et al. 2011). If the flavonoid kaempferol, a polyphenol, promotes NO bioavailability and might have the same effect as the NO donor of dietary nitrate. Kaempferol increases NO production by modulating eNOS expression and inhibiting the expression of inducible NO synthase (iNOS) (García‐Mediavilla et al. 2007; Hu et al. 2020; Rostoka et al. 2010; Xiao et al. 2009).

The improvement in mechanical efficiency is presumed to be another important mechanism. Reducing the ATP cost could lessen the cardiopulmonary load (Larsen et al. 2007). The ATP cost of skeletal muscle contraction is the total ATP consumed by the interaction between myocytes (actomyosin‐ATPase) and Ca^2+^ pumped into the sarcoplasmic reticulum (Ca^2+^‐ATPase) (Barclay, Woledge, and Curtin 2007). Quercetin, a homolog of kaempferol, suppresses Ca^2+^‐ATPase activity and enhances Ca^2+^‐release channel activity (Kim, Ohnishi, and Ikemoto 1983; Lee, Meissner, and Kim 2002). The increased sensitivity of myocytes to Ca^2+^ can positively affect the mechanical efficiency and strength of skeletal muscles (Bazzucchi et al. 2019; Kim, Ohnishi, and Ikemoto 1983; Lee, Meissner, and Kim 2002). An improvement in mechanical efficiency has been reported for the NO‐mediated mechanism (Bailey et al. 2010; Jones 2014).

Moreover, it has been suggested that the flavonoid quercetin may block adenosine receptors at the central level, which may be involved in the energetics of neurotransmitter uptake and could influence motor unit recruitment capacity, thereby contributing to mechanical efficiency and strength (Davis et al. 2009). Accordingly, kaempferol homolog may have similar functions. Like caffeine, flavonoids affect the central nervous system; their effects may be linked to positive emotions, toughness, and upsurge and can affect physical performance (Alexander 2006; Davis et al. 2003).

Kaempferol and certain polyphenols can enhance mitochondrial biogenesis via the peroxisome proliferator‐activated receptor gamma coactivator 1α and nuclear respiratory factor 2/Kelch‐like ECH‐associated protein one pathway (Alshehri et al. 2022; Beekmann et al. 2015; Hussain et al. 2022; Wood Dos Santos et al. 2018). However, because the current results were obtained using a single dose of kaempferol, they are unlikely to be due to enhanced mitochondrial biogenesis.

In the current study, when the cardiopulmonary load was reduced, the decrease in respiratory rate was more noticeable than that in heart rate. Therefore, kaempferol may stabilize and improve airway and respiratory function during exercise. A cross‐sectional study suggested that polyphenols enhance respiratory function regarding forced vital capacity and expiratory volume (Pounis et al. 2018). In addition, exercise‐induced bronchoconstriction is observed in 20%–50% of athletes and 7%–10% of the general population during ultra‐high‐intensity exercise, even in people without bronchial asthma (Parsons et al. 2013; Pigakis et al. 2022; Pongdee and Li 2013). Thus, it is assumed that bronchoconstriction occurs at a specific frequency during the highly intense exercise of the total 400‐m run. Exercise‐induced bronchoconstriction may be caused by hyperventilation, hyperosmotic environment, and increased penetration of allergens and pollutants, resulting in airway inflammation and epithelial injury (Boulet and O'Byrne 2015; Parsons et al. 2013; Pongdee and Li 2013). Kaempferol has been experimentally shown to be effective against inflammatory disorders, including bronchial asthma, because of its strong antioxidant activity and various antiallergic effects (Molitorisova et al. 2021; Rajendran et al. 2014), and it may be possible that kaempferol stabilizes airway hypersensitivity during 400‐m runs in outdoor fields.

In the current study, kaempferol intake reduced the elevation of blood markers of muscle damage in repeated 400‐m runs. Strenuous exercise increases ROS production through several pathways, leading to muscle fiber damage, eventually resulting in muscle fatigue (Peternelj and Coombes 2011; Urso 2013). The potent antioxidant and anti‐inflammatory effects of kaempferol (Chen and Chen 2013; Singh et al. 2008; Tzeng et al. 2011; Yao et al. 2019) may suppress muscle damage. Kaempferol may improve the mechanical efficiency of muscle contraction and(or) motor unit recruitment, reducing muscle stress (Davis et al. 2009).

The current results suggest that kaempferol reduces the cardiopulmonary load during ultra‐high‐intensity exercise and that kaempferol may benefit people living at high altitudes in hypoxic environments. Although environmental adaptation, such as improved respiratory function and oxygen uptake capacity, are the most important factors, the consumption of crops cultivated in the highlands could play a role. According to one survey, most kaempferol‐rich supplements are harvested from highland areas at altitudes > 1000 m (Mizokami, Akiyama, and Ikeda 2021). Environmental stresses, such as UV light exposure, increase the flavonoid content in plants (Dinkova‐Kostova 2008). In our previous study, we compared the kaempferol content in crops cultivated in lowland and highland areas and observed that crops cultivated in highland areas had greater kaempferol content (Mizokami, Akiyama, and Ikeda 2021). In addition, the kaempferol content increases in crops grown from seeds under hypoxic conditions (Mizokami, Akiyama, and Ikeda 2021). Grains containing kaempferol provided dietary support and improved the health and physical fitness of the highland residents.

One limitation of this study was its small sample size. As mentioned in the statistics section, although it is true that we were able to conduct the study with more participants than the 9 or 11 used in previous studies investigating the effects of supplement intake on exercise performance, ideally, more athletes need to be included in the study. Another limitation of this study is that while we observed a significant reduction in cardiopulmonary load during the 400 m run, we were unable to observe a corresponding improvement in race times. However, a substantial decrease in the second run time was observed after kaempferol intake, which is interesting. This supplement may improve endurance and performance in repeated competitions. Future studies need to be conducted to confirm this involving top‐level track athletes with similar times. In addition, we demonstrated the significant effects of a single intake of kaempferol. However, from a practical standpoint, it is necessary to compare the effects of a single intake with multi‐day intake in other intense exercises and various endurance sports. The strength of this study is that it is the first to observe the effects of kaempferol on exercise performance in a rigorous double‐blind trial. Additionally, consistent with previous studies, it can be unequivocally stated that no physical problems were observed because of kaempferol intake.

In conclusion, our findings showed that a single oral dose of kaempferol improved athletic and competitive ability and inhibited cardiopulmonary burden and muscle damage in 400‐m races. An increase in anaerobic metabolism did not accompany this effect. Kaempferol may serve as a useful supplement, particularly in individuals performing strenuous exercises with high oxygen demand. However, the underlying mechanism remains unclear. Thus, further research is required to elucidate the underlying mechanism and investigate its interdisciplinary utility.

## Author Contributions

**Koichi Okita:** conceptualization (equal), data curation (equal), investigation (equal), methodology (equal), visualization (equal), writing – original draft (lead), and writing – review and editing (lead). **Tsubasa Mizokami:** data curation (equal), formal analysis (equal), investigation (equal), and visualization (equal). **Minoru Akiyama:** data curation (equal), formal analysis (equal), investigation (equal), and visualization (equal). **Yasutaka Ikeda:** conceptualization (equal), data curation (equal), investigation (equal), methodology (equal), visualization (equal), and writing, review, and editing (lead).

## Conflicts of Interest

The authors declare no conflicts of interest.

## Supporting information


Figure S1.

Table S1.

Table S2.

Table S3.

Table S4.

Table S5.

Table S6.

## Data Availability

The data supporting the findings of this study are available from the corresponding author upon request.

## References

[fsn34506-bib-0001] Akiyama, M. , T. Mizokami , H. Ito , and Y. Ikeda . 2023. “A Randomized, Placebo‐Controlled Trial Evaluating the Safety of Excessive Administration of Kaempferol Aglycone.” Food Science & Nutrition 11, no. 9: 5427–5437. 10.1002/fsn3.3499. 37701215 PMC10494647

[fsn34506-bib-0002] Akiyama, M. , T. Mizokami , S. Miyamoto , and Y. Ikeda . 2022. “Kaempferol Increases Intracellular ATP Content in C(2)C(12) Myotubes Under Hypoxic Conditions by Suppressing the HIF‐1alpha Stabilization and/or by Enhancing the Mitochondrial Complex IV Activity.” Journal of Nutritional Biochemistry 103: 108949. 10.1016/j.jnutbio.2022.108949. 35122998

[fsn34506-bib-0003] Alexander, S. P. 2006. “Flavonoids as Antagonists at A1 Adenosine Receptors.” Phytotherapy Research 20, no. 11: 1009–1012. 10.1002/ptr.1975. 17006974

[fsn34506-bib-0004] Alshehri, A. S. , A. F. El‐Kott , M. S. A. El‐Gerbed , A. E. El‐Kenawy , G. M. Albadrani , and H. S. Khalifa . 2022. “Kaempferol Prevents Cadmium Chloride‐Induced Liver Damage by Upregulating Nrf2 and Suppressing NF‐κB and keap1.” Environmental Science and Pollution Research International 29, no. 10: 13917–13929. 10.1007/s11356-021-16711-3. 34599712

[fsn34506-bib-0005] Bailey, S. J. , J. Fulford , A. Vanhatalo , et al. 2010. “Dietary Nitrate Supplementation Enhances Muscle Contractile Efficiency During Knee‐Extensor Exercise in Humans.” Journal of Applied Physiology 109, no. 1: 135–148. 10.1152/japplphysiol.00046.2010. 20466802

[fsn34506-bib-0006] Barclay, C. J. , R. C. Woledge , and N. A. Curtin . 2007. “Energy Turnover for Ca^2+^ Cycling in Skeletal Muscle.” Journal of Muscle Research and Cell Motility 28, no. 4–5: 259–274. 10.1007/s10974-007-9116-7. 17882515

[fsn34506-bib-0007] Bazzucchi, I. , F. Patrizio , R. Ceci , et al. 2019. “The Effects of Quercetin Supplementation on Eccentric Exercise‐Induced Muscle Damage.” Nutrients 11, no. 1: 205. 10.3390/nu11010205. 30669587 PMC6356612

[fsn34506-bib-0008] Beekmann, K. , L. Rubió , L. H. de Haan , et al. 2015. “The Effect of Quercetin and Kaempferol Aglycones and Glucuronides on Peroxisome Proliferator‐Activated Receptor‐Gamma (PPAR‐γ).” Food & Function 6, no. 4: 1098–1107. 10.1039/c5fo00076a. 25765892

[fsn34506-bib-0009] Borg, G. A. 1982. “Psychophysical Bases of Perceived Exertion.” Medicine and Science in Sports and Exercise 14, no. 5: 377–381. 7154893

[fsn34506-bib-0010] Boulet, L. P. , and P. M. O'Byrne . 2015. “Asthma and Exercise‐Induced Bronchoconstriction in Athletes.” New England Journal of Medicine 372, no. 7: 641–648. 10.1056/NEJMra1407552. 25671256

[fsn34506-bib-0011] Caciano, S. L. , C. L. Inman , E. E. Gockel‐Blessing , and E. P. Weiss . 2015. “Effects of Dietary Acid Load on Exercise Metabolism and Anaerobic Exercise Performance.” Journal of Sports Science and Medicine 14, no. 2: 364–371. 25983586 PMC4424466

[fsn34506-bib-0012] Calderon‐Montano, J. M. , E. Burgos‐Moron , C. Perez‐Guerrero , and M. Lopez‐Lazaro . 2011. “A Review on the Dietary Flavonoid Kaempferol.” Mini Reviews in Medicinal Chemistry 11, no. 4: 298–344. 10.2174/138955711795305335. 21428901

[fsn34506-bib-0013] Chen, A. Y. , and Y. C. Chen . 2013. “A Review of the Dietary Flavonoid, Kaempferol on Human Health and Cancer Chemoprevention.” Food Chemistry 138, no. 4: 2099–2107. 10.1016/j.foodchem.2012.11.139. 23497863 PMC3601579

[fsn34506-bib-0014] Davis, J. M. , E. A. Murphy , M. D. Carmichael , and B. Davis . 2009. “Quercetin Increases Brain and Muscle Mitochondrial Biogenesis and Exercise Tolerance.” American Journal of Physiology. Regulatory, Integrative and Comparative Physiology 296, no. 4: R1071–R1077. 10.1152/ajpregu.90925.2008. 19211721

[fsn34506-bib-0015] Davis, J. M. , Z. Zhao , H. S. Stock , K. A. Mehl , J. Buggy , and G. A. Hand . 2003. “Central Nervous System Effects of Caffeine and Adenosine on Fatigue.” American Journal of Physiology‐Regulatroy, Integrative and Comparative Physiology 284, no. 2: R399–R404. 10.1152/ajpregu.00386.2002. 12399249

[fsn34506-bib-0016] Dinkova‐Kostova, A. T. 2008. “Phytochemicals as Protectors Against Ultraviolet Radiation: Versatility of Effects and Mechanisms.” Planta Medica 74, no. 13: 1548–1559. 10.1055/s-2008-1081296. 18696411

[fsn34506-bib-0017] d'Unienville, N. M. A. , H. T. Blake , A. M. Coates , A. M. Hill , M. J. Nelson , and J. D. Buckley . 2021. “Effect of Food Sources of Nitrate, Polyphenols, L‐arginine and L‐citrulline on Endurance Exercise Performance: A Systematic Review and Meta‐Analysis of Randomised Controlled Trials.” Journal of the International Society of Sports Nutrition 18, no. 1: 76. 10.1186/s12970-021-00472-y. 34965876 PMC8715640

[fsn34506-bib-0018] García‐Mediavilla, V. , I. Crespo , P. S. Collado , et al. 2007. “The Anti‐Inflammatory Flavones Quercetin and Kaempferol Cause Inhibition of Inducible Nitric Oxide Synthase, Cyclooxygenase‐2 and Reactive C‐Protein, and Down‐Regulation of the Nuclear Factor KappaB Pathway in Chang Liver Cells.” European Journal of Pharmacology 557, no. 2–3: 221–229. 10.1016/j.ejphar.2006.11.014. 17184768

[fsn34506-bib-0019] Hu, W. H. , H. Y. Wang , Y. T. Xia , et al. 2020. “Kaempferol, a Major Flavonoid in Ginkgo Folium, Potentiates Angiogenic Functions in Cultured Endothelial Cells by Binding to Vascular Endothelial Growth Factor.” Frontiers in Pharmacology 11: 526. 10.3389/fphar.2020.00526. 32410995 PMC7198864

[fsn34506-bib-0020] Hussain, Y. , H. Khan , K. F. Alsharif , A. Hayat Khan , M. Aschner , and L. Saso . 2022. “The Therapeutic Potential of Kaemferol and Other Naturally Occurring Polyphenols Might Be Modulated by Nrf2‐ARE Signaling Pathway: Current Status and Future Direction.” Molecules 27, no. 13: 4145. 10.3390/molecules27134145. 35807387 PMC9268049

[fsn34506-bib-0021] Imran, M. , B. Salehi , J. Sharifi‐Rad , et al. 2019. “Kaempferol: A Key Emphasis to Its Anticancer Potential.” Molecules 24, no. 12: 2277. 10.3390/molecules24122277. 31248102 PMC6631472

[fsn34506-bib-0022] Jones, A. M. 2014. “Dietary Nitrate Supplementation and Exercise Performance.” Sports Medicine 44, no. Suppl 1: S35–S45. 10.1007/s40279-014-0149-y. 24791915 PMC4008816

[fsn34506-bib-0023] Jones, A. M. , A. Vanhatalo , D. R. Seals , M. J. Rossman , B. Piknova , and K. L. Jonvik . 2021. “Dietary Nitrate and Nitric Oxide Metabolism: Mouth, Circulation, Skeletal Muscle, and Exercise Performance.” Medicine and Science in Sports and Exercise 53, no. 2: 280–294. 10.1249/mss.0000000000002470. 32735111

[fsn34506-bib-0024] Kelly, J. , A. Vanhatalo , D. P. Wilkerson , L. J. Wylie , and A. M. Jones . 2013. “Effects of Nitrate on the Power‐Duration Relationship for Severe‐Intensity Exercise.” Medicine and Science in Sports and Exercise 45, no. 9: 1798–1806. 10.1249/MSS.0b013e31828e885c. 23475164

[fsn34506-bib-0025] Kim, D. H. , S. T. Ohnishi , and N. Ikemoto . 1983. “Kinetic Studies of Calcium Release From Sarcoplasmic Reticulum In Vitro.” Journal of Biological Chemistry 258, no. 16: 9662–9668. 6309780

[fsn34506-bib-0026] Kinugawa, S. , Z. Wang , P. M. Kaminski , et al. 2005. “Limited Exercise Capacity in Heterozygous Manganese Superoxide Dismutase Gene‐Knockout Mice: Roles of Superoxide Anion and Nitric Oxide.” Circulation 111, no. 12: 1480–1486. 10.1161/01.CIR.0000159261.11520.63. 15781740

[fsn34506-bib-0027] Larsen, F. J. , T. A. Schiffer , S. Borniquel , et al. 2011. “Dietary Inorganic Nitrate Improves Mitochondrial Efficiency in Humans.” Cell Metabolism 13, no. 2: 149–159. 10.1016/j.cmet.2011.01.004. 21284982

[fsn34506-bib-0028] Larsen, F. J. , E. Weitzberg , J. O. Lundberg , and B. Ekblom . 2007. “Effects of Dietary Nitrate on Oxygen Cost During Exercise.” Acta Physiologica (Oxford, England) 191, no. 1: 59–66. 10.1111/j.1748-1716.2007.01713.x. 17635415

[fsn34506-bib-0029] Lee, E. H. , G. Meissner , and D. H. Kim . 2002. “Effects of Quercetin on Single Ca(2+) Release Channel Behavior of Skeletal Muscle.” Biophysical Journal 82, no. 3: 1266–1277. 10.1016/S0006-3495(02)75483-0. 11867444 PMC1301930

[fsn34506-bib-0030] Limmer, M. , A. D. Eibl , and P. Platen . 2018. “Enhanced 400‐m Sprint Performance in Moderately Trained Participants by a 4‐Day Alkalizing Diet: A Counterbalanced, Randomized Controlled Trial.” Journal of the International Society of Sports Nutrition 15, no. 1: 25. 10.1186/s12970-018-0231-1. 29855319 PMC5984464

[fsn34506-bib-0031] Luo, C. , X. Xu , X. Wei , et al. 2019. “Natural Medicines for the Treatment of Fatigue: Bioactive Components, Pharmacology, and Mechanisms.” Pharmacological Research 148: 104409. 10.1016/j.phrs.2019.104409. 31446039

[fsn34506-bib-0032] Mizokami, T. , M. Akiyama , and Y. Ikeda . 2021. “Kaempferol as a Phytochemical Increases ATP Content in C2C12 Myotubes Under Hypoxic Conditions.” Journal of Functional Foods 85: 104510. 10.1016/j.jff.2021.104510.

[fsn34506-bib-0033] Molitorisova, M. , M. Sutovska , I. Kazimierova , et al. 2021. “The Anti‐Asthmatic Potential of Flavonol Kaempferol in an Experimental Model of Allergic Airway Inflammation.” European Journal of Pharmacology 891: 173698. 10.1016/j.ejphar.2020.173698. 33129789

[fsn34506-bib-0034] Parsons, J. P. , T. S. Hallstrand , J. G. Mastronarde , et al. 2013. “An Official American Thoracic Society Clinical Practice Guideline: Exercise‐Induced Bronchoconstriction.” American Journal of Respiratory and Critical Care Medicine 187, no. 9: 1016–1027. 10.1164/rccm.201303-0437ST. 23634861

[fsn34506-bib-0035] Pawlak‐Chaouch, M. , J. Boissière , F. X. Gamelin , G. Cuvelier , S. Berthoin , and J. Aucouturier . 2016. “Effect of Dietary Nitrate Supplementation on Metabolic Rate During Rest and Exercise in Human: A Systematic Review and a Meta‐Analysis.” Nitric Oxide 53: 65–76. 10.1016/j.niox.2016.01.001. 26772523

[fsn34506-bib-0036] Peternelj, T. T. , and J. S. Coombes . 2011. “Antioxidant Supplementation During Exercise Training: Beneficial or Detrimental?” Sports Medicine 41, no. 12: 1043–1069. 10.2165/11594400-000000000-00000. 22060178

[fsn34506-bib-0037] Pigakis, K. M. , V. T. Stavrou , I. Pantazopoulos , Z. Daniil , A. K. Kontopodi , and K. Gourgoulianis . 2022. “Exercise‐Induced Bronchospasm in Elite Athletes.” Cureus 14, no. 1: e20898. 10.7759/cureus.20898. 35145802 PMC8807463

[fsn34506-bib-0038] Pongdee, T. , and J. T. Li . 2013. “Exercise‐Induced Bronchoconstriction.” Annals of Allergy, Asthma & Immunology 110, no. 5: 311–315. 10.1016/j.anai.2013.02.002. 23621999

[fsn34506-bib-0039] Pounis, G. , A. Arcari , S. Costanzo , et al. 2018. “Favorable Association of Polyphenol‐Rich Diets With Lung Function: Cross‐Sectional Findings From the Moli‐Sani Study.” Respiratory Medicine 136: 48–57. 10.1016/j.rmed.2017.12.007. 29501246

[fsn34506-bib-0040] Rajendran, P. , T. Rengarajan , N. Nandakumar , R. Palaniswami , Y. Nishigaki , and I. Nishigaki . 2014. “Kaempferol, a Potential Cytostatic and Cure for Inflammatory Disorders.” European Journal of Medicinal Chemistry 86: 103–112. 10.1016/j.ejmech.2014.08.011. 25147152

[fsn34506-bib-0041] Rizza, S. , R. Muniyappa , M. Iantorno , et al. 2011. “Citrus Polyphenol Hesperidin Stimulates Production of Nitric Oxide in Endothelial Cells While Improving Endothelial Function and Reducing Inflammatory Markers in Patients With Metabolic Syndrome.” Journal of Clinical Endocrinology and Metabolism 96, no. 5: E782–E792. 10.1210/jc.2010-2879. 21346065 PMC3085197

[fsn34506-bib-0042] Rostoka, E. , L. Baumane , S. Isajevs , et al. 2010. “Effects of Kaempferol and Myricetin on Inducible Nitric Oxide Synthase Expression and Nitric Oxide Production in Rats.” Basic & Clinical Pharmacology & Toxicology 106, no. 6: 461–466. 10.1111/j.1742-7843.2009.00526.x. 20088846

[fsn34506-bib-0043] Serreli, G. , and M. Deiana . 2023. “Role of Dietary Polyphenols in the Activity and Expression of Nitric Oxide Synthases: A Review.” Antioxidants (Basel) 12, no. 1: 147. 10.3390/antiox12010147. 36671009 PMC9854440

[fsn34506-bib-0044] Singh, R. , B. Singh , S. Singh , N. Kumar , S. Kumar , and S. Arora . 2008. “Anti‐Free Radical Activities of Kaempferol Isolated From *Acacia nilotica* (L.) Willd. Ex. Del.” Toxicology In Vitro 22, no. 8: 1965–1970. 10.1016/j.tiv.2008.08.007. 18805478

[fsn34506-bib-0045] Stoclet, J. C. , T. Chataigneau , M. Ndiaye , et al. 2004. “Vascular Protection by Dietary Polyphenols.” European Journal of Pharmacology 500, no. 1–3: 299–313. 10.1016/j.ejphar.2004.07.034. 15464042

[fsn34506-bib-0046] Tzeng, C. W. , F. L. Yen , T. H. Wu , et al. 2011. “Enhancement of Dissolution and Antioxidant Activity of Kaempferol Using a Nanoparticle Engineering Process.” Journal of Agricultural and Food Chemistry 59, no. 9: 5073–5080. 10.1021/jf200354y. 21417334

[fsn34506-bib-0047] Urso, M. L. 2013. “Anti‐Inflammatory Interventions and Skeletal Muscle Injury: Benefit or Detriment?” Journal of Applied Physiology 115, no. 6: 920–928. 10.1152/japplphysiol.00036.2013. 23539314

[fsn34506-bib-0048] Wang, J. , X. Fang , L. Ge , et al. 2018. “Antitumor, Antioxidant and Anti‐Inflammatory Activities of Kaempferol and Its Corresponding Glycosides and the Enzymatic Preparation of Kaempferol.” PLoS One 13, no. 5: e0197563. 10.1371/journal.pone.0197563. 29771951 PMC5957424

[fsn34506-bib-0049] Wood Dos Santos, T. , Q. Cristina Pereira , L. Teixeira , A. Gambero , J. A. Villena , and M. Lima Ribeiro . 2018. “Effects of Polyphenols on Thermogenesis and Mitochondrial Biogenesis.” International Journal of Molecular Sciences 19, no. 9: 2757. 10.3390/ijms19092757. 30217101 PMC6164046

[fsn34506-bib-0050] Xiao, H. B. , F. Jun , X. Y. Lu , X. J. Chen , T. Chao , and Z. L. Sun . 2009. “Protective Effects of Kaempferol Against Endothelial Damage by an Improvement in Nitric Oxide Production and a Decrease in Asymmetric Dimethylarginine Level.” European Journal of Pharmacology 616, no. 1–3: 213–222. 10.1016/j.ejphar.2009.06.022. 19549512

[fsn34506-bib-0051] Yao, X. , H. Jiang , Y. NanXu , X. Piao , Q. Gao , and N. H. Kim . 2019. “Kaempferol Attenuates Mitochondrial Dysfunction and Oxidative Stress Induced by H(2)O(2) During Porcine Embryonic Development.” Theriogenology 135: 174–180. 10.1016/j.theriogenology.2019.06.013. 31226607

[fsn34506-bib-0052] Zaynab, M. , M. Fatima , S. Abbas , et al. 2018. “Role of Secondary Metabolites in Plant Defense Against Pathogens.” Microbial Pathogenesis 124: 198–202. 10.1016/j.micpath.2018.08.034. 30145251

