# Supplementary material for: Kaempferol Reduces Cardiopulmonary Load and Muscular Damage in Repeated 400‐m Sprints: A Double‐Blind, Randomized, Placebo‐Controlled Trial
Source: Food Sci Nutr. 2024 Oct 14;12(11):9458–68. doi: 10.1002/fsn3.4506 (PMC11606868; doi:10.1002/fsn3.4506)
Supplement: Supplementary file 2 — Table S1. [file FSN3-12-9458-s006.pdf]

Supplementary Table 1. Blood kaempferol concentration.

Group	Blood kaempferol concentration (μM)				
	Sampling points				
	1st run		2nd run		
	pre	post	pre	post	3 h
Placebo	0.007 ± 0.017	0.007 ± 0.017	0.006 ± 0.014	0.007 ± 0.016	0.005 ± 0.014
Active	0.253 ± 0.073	0.239 ± 0.072	0.195 ± 0.068	0.201 ± 0.070	0.100 ± 0.058

Active means a 10 mg kaempferol-containing capsule. Data are presented as mean ± SD.
