# Supplementary material for: Kaempferol Reduces Cardiopulmonary Load and Muscular Damage in Repeated 400‐m Sprints: A Double‐Blind, Randomized, Placebo‐Controlled Trial
Source: Food Sci Nutr. 2024 Oct 14;12(11):9458–68. doi: 10.1002/fsn3.4506 (PMC11606868; doi:10.1002/fsn3.4506)
Supplement: Supplementary file 3 — Table S2. [file FSN3-12-9458-s002.pdf]

Supplementary Table 2. Twenty-four-hour urinary kaempferol excretion and excretion rates.

Group	Excretion (mg)	Excretion rate (%)
Placebo	0.005 ± 0.017	–
Active	0.336 ± 0.194	3.36 ± 1.94

Active means a 10 mg kaempferol-containing capsule. Data are presented as mean ± SD.
