# Supplementary material for: Kaempferol Reduces Cardiopulmonary Load and Muscular Damage in Repeated 400‐m Sprints: A Double‐Blind, Randomized, Placebo‐Controlled Trial
Source: Food Sci Nutr. 2024 Oct 14;12(11):9458–68. doi: 10.1002/fsn3.4506 (PMC11606868; doi:10.1002/fsn3.4506)
Supplement: Supplementary file 4 — Table S3. [file FSN3-12-9458-s005.pdf]

Supplementary Table 3. Stride width during 400-m runs.

Runs	Group	Stride width (m)							
		Points							
		0–50 m	50–100 m	100–150 m	150–200 m	200–250 m	250–300 m	300–350 m	350–400 m
1st run	Placebo	1.96 ± 0.17	2.12 ± 0.22	2.01 ± 0.22	1.96 ± 0.22	1.93 ± 0.23	1.87 ± 0.23	1.82 ± 0.20	1.82 ± 0.24
	Active	1.88 ± 0.17	2.09 ± 0.22	2.02 ± 0.22	1.94 ± 0.22	1.93 ± 0.22	1.85 ± 0.22	1.82 ± 0.21	1.82 ± 0.24
2nd run	Placebo	1.89 ± 0.17	2.12 ± 0.21	2.01 ± 0.21	1.96 ± 0.21	1.95 ± 0.24	1.88 ± 0.21	1.87 ± 0.24	1.86 ± 0.22
	Active	1.90 ± 0.15	2.10 ± 0.21	2.03 ± 0.20	1.97 ± 0.19	1.95 ± 0.20	1.88 ± 0.21	1.86 ± 0.18	1.84 ± 0.18

Active means a 10 mg kaempferol-containing capsule. Data are presented as mean ± SD.
