# Supplementary material for: Kaempferol Reduces Cardiopulmonary Load and Muscular Damage in Repeated 400‐m Sprints: A Double‐Blind, Randomized, Placebo‐Controlled Trial
Source: Food Sci Nutr. 2024 Oct 14;12(11):9458–68. doi: 10.1002/fsn3.4506 (PMC11606868; doi:10.1002/fsn3.4506)
Supplement: Supplementary file 5 — Table S4. [file FSN3-12-9458-s004.pdf]

Supplementary Table 4. Pitch during 400-m runs.

Runs	Group	Pitch (steps/sec)							
		Points							
		0–50 m	50–100 m	100–150 m	150–200 m	200–250 m	250–300 m	300–350 m	350–400 m
1st run	Placebo	3.74 ± 0.33	3.84 ± 0.32	3.72 ± 0.28	3.56 ± 0.19	3.47 ± 0.18	3.40 ± 0.17	3.36 ± 0.14	3.26 ± 0.15
	Active	3.76 ± 0.34	3.87 ± 0.34	3.67 ± 0.31	3.54 ± 0.25	3.43 ± 0.22	3.38 ± 0.17	3.36 ± 0.14	3.22 ± 0.10
2nd run	Placebo	3.73 ± 0.25	3.79 ± 0.29	3.62 ± 0.25	3.54 ± 0.20	3.42 ± 0.20	3.38 ± 0.18	3.38 ± 0.16	3.30 ± 0.15
	Active	3.75 ± 0.29	3.81 ± 0.29	3.69 ± 0.29	3.59 ± 0.22	3.46 ± 0.21	3.38 ± 0.15	3.36 ± 0.18	3.30 ± 0.13

Active means a 10 mg kaempferol-containing capsule. Data are presented as mean ± SD.
