# Supplementary material for: Kaempferol Reduces Cardiopulmonary Load and Muscular Damage in Repeated 400‐m Sprints: A Double‐Blind, Randomized, Placebo‐Controlled Trial
Source: Food Sci Nutr. 2024 Oct 14;12(11):9458–68. doi: 10.1002/fsn3.4506 (PMC11606868; doi:10.1002/fsn3.4506)
Supplement: Supplementary file 6 — Table S5. [file FSN3-12-9458-s001.pdf]

Supplementary Table 5. Changes in the blood marker levels for exercise metabolism (absolute values).

Variables	Group	Sampling points				
		1st run		2nd run		
		pre	post	pre	post	3 h
Lactate (mg·dL^{-1})	Placebo	11.4 ± 3.1	146.6 ± 19.8§ (§P< 0.0001)	18.7 ± 4.6	133.8 ± 21.1§‖ (§P< 0.0001) (‖P< 0.0001)	9.9 ± 2.0
	Active	11.8 ± 3.7	148.0 ± 19.8§ (§P< 0.0001)	15.8 ± 5.9	143.7 ± 24.7§‖ (§P< 0.0001) (‖P< 0.0001)	8.5 ± 2.6
Pyruvate (mg·dL^{-1})	Placebo	0.82 ± 0.23	3.09 ± 0.78§ (§P< 0.0001)	0.94 ± 0.26	2.98 ± 0.76§‖ (§P< 0.0001) (‖P< 0.0001)	0.61 ± 0.12
	Active	0.69 ± 0.14	2.90 ± 0.77§ (§P< 0.0001)	0.76 ± 0.28	2.91 ± 0.90§‖ (§P< 0.0001) (‖P< 0.0001)	0.51 ± 0.12

Active means a 10 mg kaempferol-containing capsule. Data are presented as mean ± SD (§P<0.05 vs pre 1st run, ‖P<0.05 vs pre 2nd run). Mixed model for crossover design.
