# Supplementary material for: Kaempferol Reduces Cardiopulmonary Load and Muscular Damage in Repeated 400‐m Sprints: A Double‐Blind, Randomized, Placebo‐Controlled Trial
Source: Food Sci Nutr. 2024 Oct 14;12(11):9458–68. doi: 10.1002/fsn3.4506 (PMC11606868; doi:10.1002/fsn3.4506)
Supplement: Supplementary file 7 — Table S6. [file FSN3-12-9458-s003.pdf]

Supplementary Table 6. Changes in the blood marker levels for muscle damage (absolute values).

Variables	Group	Sampling points				
		1st run		2nd run		
		pre	post	pre	post	3 h
Myoglobin (ng·mL^{-1})	Placebo	51.0 ± 18.7	62.6 ± 26.2¶<br>(¶*P*=0.0622)	92.1 ± 45.5§<br>(§*P*< 0.0001)	100.7 ± 49.7§<br>(§*P*< 0.0001)	102.5 ± 46.8§<br>(§*P*< 0.0001)
	Active	51.6 ± 13.8	58.2 ± 17.2	80.5 ± 28.2§<br>(§*P*< 0.0001)	88.0 ± 31.9§<br>(§*P*< 0.0001)	88.2 ± 38.3§†<br>(§*P*< 0.0001)<br>(†*P=0.0797*)
CPK (U·L-^{1})	Placebo	312.4 ± 248.8	374.0 ± 229.1§<br>(§*P*< 0.0001)	339.8 ± 270.1§<br>(§*P*= 0.0123)	399.2 ± 315.1§<br>(§*P*< 0.0001)	359.5 ± 262.8§<br>(§*P*< 0.0001)
	Active	287.6 ± 108.9	342.0 ± 126.2§<br>(§*P*< 0.0001)	311.9 ± 119.7§<br>(§*P*= 0.0123)	364.1 ± 141.0§<br>(§*P*< 0.0001)	327.7 ± 123.1§<br>(§*P*< 0.0001)
AST (U·L^{-1})	Placebo	25.6 ± 8.4	31.0 ± 10.5§<br>(§*P*< 0.0001)	27.1 ± 9.1§<br>(§*P*= 0.0044)	32.0 ± 10.3§<br>(§*P*< 0.0001)	26.3 ± 8.2§<br>(§*P*< 0.0001)
	Active	23.6 ± 5.5	28.2 ± 6.3§<br>(§*P*< 0.0001)	24.8 ± 6.2§<br>(§*P*= 0.0253)	28.8 ± 6.9§†<br>(§*P*< 0.0001)<br>(†*P*= 0.0961)	24.3 ± 5.5§<br>(§*P*< 0.0001)
ALT (U·L^{-1})	Placebo	25.8 ± 13.4	29.6 ± 15.4§<br>(§*P*< 0.0001)	26.3 ± 13.7	29.8 ± 15.0§<br>(§*P*< 0.0001)	25.3 ± 12.3
	Active	20.6 ± 7.6	23.6 ± 8.5§<br>(§*P*< 0.0001)	21.4 ± 7.6	23.8 ± 8.8§†<br>(§*P*< 0.0001)<br>(†*P*=0.0741)	20.6 ± 6.9
LDH (U/L)	Placebo	173.1 ± 22.3	214.1 ± 24.9§<br>(§*P*< 0.0001)	185.0 ± 23.1§<br>(§*P*= 0.0445)	224.2 ± 31.3§<br>(§*P*< 0.0001)	184.3 ± 21.4§<br>(§*P*= 0.0370)
	Active	173.1 ± 22.0	211.4 ± 19.3§<br>(§*P*< 0.0001)	185.3 ± 24.6§<br>(§*P*= 0.0027)	222.5 ± 27.0§<br>(§*P*< 0.0001)	186.4 ± 20.1§<br>(§*P*< 0.0001)

Active means a 10 mg kaempferol-containing capsule. CPK, creatine phosphokinase; AST, aspartate transaminase; ALT, alanine aminotransferase; LDH, lactate dehydrogenase. Data are presented as mean ± SD (¶P<0.1 vs pre 1st run, §P<0.05 vs pre 1st run, †P<0.1 vs Placebo). Mixed model for crossover design.
